# Aluminium Ammonium Sulfate and Aluminium Potassium Sulfate (Food Additives)

**DOI:** 10.14252/foodsafetyfscj.D-19-00015

**Published:** 2019-09-30

**Authors:** Food Safety Commission of Japan

## Abstract

Food Safety Commission of Japan (FSCJ) conducted a risk assessment of aluminium ammonium sulfate and aluminium potassium sulfate. This evaluation was requested from Ministry of Health, Labour and Welfare (MHLW) to revise the standards for use of additives. Aluminium ammonium sulfate and aluminium potassium sulfate as additives are assumed reasonably to behave as ions after dissociation, such as aluminium, ammonium, potassium, and sulfate ions, in digestive tract prior to their absorption. FSCJ thus evaluated the safety of aluminium ammonium sulfate and aluminium potassium sulfate used as additives, in considering the substances that are composed of ammonium ion, sulfate ion, potassium ion and aluminium ion. FSCJ concluded that there were no safety concerns of sulfate, ammonium and potassium ions as the use of aluminium ammonium sulfate and aluminium potassium sulfate for food additives. FSCJ specified the lowest no-observed-adverse-effect level (NOAEL) of 30 mg/kg bw/day for aluminium ion based on the reproductive developmental toxicity studies in rats. FSCJ also recognized no carcinogenicity of aluminium additives. FSCJ judged no clear relationship of dietary intake of aluminium with the influences on the bone, mainly due to the insufficient amounts of evidence. FSCJ judged no sufficient evidence to indicate a causal relationship between dietary intake of aluminium and neurological diseases including Alzheimer’s disease. FSCJ confirmed that no human data exist to indicate the clear association of the dietary intake with human health effects of aluminium. FSCJ specified this metal (Al) to be 1.0 mg/kg bw/week for the children (1 to 6 years) and 0.57 mg/kg bw/week for the general population. A safety factor of 100 was applied to the NOAEL of 30 mg/kg bw/day obtained in a developmental toxicity study in rats. Converting the value thus obtained to the aluminium intake per a week, FSCJ established a tolerable weekly intake (TWI) of 2.1 mg/kg bw/week (as Al) for aluminium.

## Conclusion in Brief

Food Safety Commission of Japan (FSCJ) conducted a risk assessment of aluminium ammonium sulfate and aluminium potassium sulfate. This evaluation was requested from Ministry of Health, Labour and Welfare (MHLW) to revise the standards for use of additives. These include the substances in Table 1. Both aluminium ammonium sulfate and aluminium potassium sulfate are additives used in baking powder and also in agents for food producing and processing.

**Table 1.** Aluminium ammonium sulfate and aluminium potassium sulfate

**Aluminium ammonium sulfate**	CAS No. 7784-26-1 as aluminium ammonium sulfate dodecahydrate
	CAS No. 7784-25-0 as aluminium ammonium sulfate (anhydride)
**Aluminium potassium sulfate**	CAS No. 7784-24-9 as aluminium potassium sulfate dodecahydrate
	CAS No. 10043-67-1 as aluminium potassium sulfate (anhydride)

The data used in the assessment include pharmacokinetics (Absorption, Distribution, Metabolism, Excretion (ADME)), genotoxicity, repeated dose toxicity, carcinogenicity, and reproductive and developmental toxicity. Epidemiological studies and case reports of aluminium are also included.

Aluminium ammonium sulfate and aluminium potassium sulfate as additives are assumed reasonably to behave as ions after dissociation, such as aluminium, ammonium, potassium, and sulfate ions, in digestive tract prior to their absorption. Therefore, the data on the related substances including these specific ions as well as aluminium ammonium sulfate and aluminium potassium sulfate are added for the evaluation. FSCJ thus evaluated the safety of aluminium ammonium sulfate and aluminium potassium sulfate used as additives, in considering the substances that are composed of ammonium ion, sulfate ion, potassium ion and aluminium ion.

## 1. Ammonium, Potassium, and Sulfate Ions

As for ammonium ion, the risk assessment report^1^^)^ of an additive, ammonium isovalerate, described that human digestive tracts produced ammonia through food consumption up to 10 mg/day in the duodenum and ca. 3 g/day in the colon. The ammonia thus, produced in digestive tract, is mostly absorbed to enter into the portal circulation. In healthy humans, ammonium ion is readily converted to urea in the liver and then excreted in urine through kidney.

The amount of ammonia generated in the human body after ingestion of “aluminium ammonium sulfate” is considered to be within amount ranges of ammonia produced through food consumption. The ammonia generated is thus metabolized in the human body in the manner similar to the ammonia produced through food consumption. FSCJ decided, therefore, not to conduct evaluation of ADME and toxicity of ammonia (ammonium ions) in this assessment.

ADME and toxicity of sulfate and potassium ions were already evaluated during the risk assessment of additives; potassium sulfate^2^^)^ and zinc sulfate^3^^)^. Both ions raised no concerns relevant to human health. Since no findings on the safety concerns of these ions were newly obtained, FSCJ decided not to evaluate again the ADME and toxicity of sulfate and potassium ions.

Hence, FSCJ concluded that there were no safety concerns of sulfate, ammonium and potassium ions as the use of aluminium ammonium sulfate and aluminium potassium sulfate for food additives.

## 2. Aluminium Ion

The national and international organizations recently conducted risk assessment of aluminium as additives and as contaminants in food indistinguishably. In consistent with this, FSCJ assessed intake of aluminium as additives and contaminants for the assessment of aluminium as ions.

### (1) ADME

Among aluminum salts, absorption rate of aluminium citrate is relatively higher than the other aluminium salts based on the evaluation of various findings on ADME of aluminium compounds. Most of aluminium absorbed is rapidly excreted from the body. However, parts of aluminium distributed in the bones and other tissues had relatively long half-lives, thus posing a potential accumulation in the body. Among the available data on different oral-administration methods (gavage, feeding and drinking), there are no substantial differences in the pharmacokinetic profiles. FSCJ evaluated aluminium in a way keeping these points.

### (2) Toxicity

Two different aluminium salts are used as additives, aluminium ammonium sulfate, and aluminium potassium sulfate. No positive results are obtained in Ames test on aluminium compounds including both the additives. Some aluminium salts induce DNA damages in comet assay and chromosomal aberration. The cells with the damaged DNA are removed selectively through apoptotic mechanism and the chromosomal aberration is also caused through rather indirect genotoxic mechanisms. FSCJ thus judged aluminium contained in these additives has no genotoxicity relevant to human health.

FSCJ evaluated the data on acute toxicity, repeated dose toxicity, reproductive and developmental toxicity, and other toxicities of aluminium compounds including both the additives. As the results, FSCJ specified the lowest no-observed-adverse-effect level (NOAEL) of 30 mg/kg bw/day for aluminium ion based on decreased bodyweight gain and effects on the kidney in male offspring observed in the reproductive developmental toxicity studies in rats by Semple^4^^)^ and Poirier et al.^5^^)^.

While changes on the lipid metabolism in a 13-week dietary toxicity study (Kawasaki et al.)^6^^)^ and hyperplasia of mucosal epithelium of urinary bladder in a 90-day dietary toxicity study (Cho et al.)^7^^)^ are reported, these effects have not been observed in the other related studies. FSCJ thus did not judge these effects to be aluminum-associated toxicity. Effects on the nervous system were reported in rats after the six-month oral exposure through drinking water (Somova & Khan^8^^)^, Somova et al.^9^^)^, and Sethi et al.^10^^)^). No toxicological details of the effects are, however, clarified and also the relevance of the pathological findings to humans remains uncertain. Moreover, knowledge for a causal relationship between exposure to aluminium through foods consumption and neurological disease including Alzheimer’s disease are limited to be evaluated. Therefore, FSCJ recognized that these neurological findings in rats are not of concerns for food safety in humans. Decreases in hormonal levels are detected in two of 120-day oral exposure tests through drinking water in rats (Sun et al.^11^^)^ and Wang^12^^)^). The effects are unlikely to be applicable to human situations due to the lack of detectable endocrine toxicity in humans.

As the results, FSCJ specified the lowest NOAEL of 30 mg/kg bw/day for aluminium ion based on the reproductive developmental toxicity studies in rats by Semple^4^^)^ and Poirier et al.^5^^)^.

FSCJ also recognized no carcinogenicity of aluminium additives.

FSCJ evaluated aluminum-related findings in humans as follows.

Association of aluminium intake with clinical manifestation has been reported for the effects on the bones, neurological diseases including Alzheimer’s disease and dialysis encephalopathy syndrome (DES). DES is, however, attributed to aluminium intake through parenteral routes.

FSCJ evaluated effects of aluminium intake on the bone based on the following findings. Several reports are available for the effects on the bone due to an inhibition of the absorption of phosphoric acid in the digestive tracts under a high-dose administration of antacids, and for the effects of aluminium intake through parenteral nutrition in neonates. Some reports showed, however, the lack of an association between aluminium level in the bone and hip fracture risks in the elderly. On the basis of these findings, FSCJ judged no clear relationship of dietary intake of aluminium with the influences on the bone mainly due to the insufficient amounts of evidence.

FSCJ evaluated potential association between aluminium intake and neurological diseases including Alzheimer’s disease based on the reported findings including the case reports published after the evaluation by Joint FAO/WHO Expert Committee on Food Additives (JECFA) in 2011. The results are controversial among the epidemiological studies; some suggest potential association between aluminium intake and Alzheimer’s disease while others denied. These studies estimated aluminium intake through either drinking water, foods or medicinal products as an exposure route without considering other possible routes. In addition, the underlying biological-mechanism of this issue remains obscure. FSCJ thus judged no sufficient evidence to indicate a causal relationship between dietary intake of aluminium and neurological diseases including Alzheimer’s disease.

FSCJ confirmed that no human data exist to indicate the clear association of the dietary intake with human health effects of aluminium.

### (3) Estimated Intake

FSCJ estimated intake of aluminium originated from foods after the enforcement of the prospective amendment of standards for use of aluminium ammonium sulfate and aluminium potassium sulfate as additives. FSCJ specified this metal (Al) to be 1.0 mg/kg bw/week for the children (1 to 6 years) and 0.57 mg/kg bw/week for the general population, although the part of the data was derived from the population over 20 years. Considering the additional intakes of aluminium derived from food-contact materials (Apparatus and Containers/Packages), and tap water, FSCJ specified the estimated intakes to be 1.2 mg/kg bw/week for children and 0.69 mg/kg bw/week for the general population.

### (4) The Risk Assessment

FSCJ recognized the necessity to specify an upper limit of intake for aluminium due to both from the additives and contaminants.

Regarding the health-based guidance value for aluminium, JECFA and EFSA applied tolerable-weekly-intake (TWI). Both organizations evaluated whole aluminium derived from both additives and contaminants.

Therefore, FSCJ adopted a TWI as the health-based guidance value for the risk assessment of aluminium.

A safety factor of 100 was applied to the NOAEL of 30 mg/kg bw/day obtained in a developmental toxicity study in rats. Converting the value thus obtained to the aluminium intake per a week, FSCJ established a TWI of 2.1 mg/kg bw/week (as Al) for aluminium.
